# LONELINESS AND SOCIAL ISOLATION IN THE COVID ERA

**DOI:** 10.1093/geroni/igae098.1656

**Published:** 2024-12-31

**Authors:** Solveig Cunningham, Jo Hale

**Affiliations:** Emory University, Atlanta, Georgia, United States; University of St. Andrews, St. Andrews, Scotland, United Kingdom

## Abstract

Social isolation and loneliness diminish health and wellbeing and may have been exacerbated during the Covid-19 lockdown. We examined the relationship between social isolation and loneliness among U.S. older adults during the Covid-19 pandemic, when social isolation was high due to transmission reduction measures. Data are from the National Health and Aging Trends Survey (NHATS), representative of the age 70+ Medicare beneficiary population in the United States. Prior to the pandemic, one third of older adults experienced loneliness at least on some days. One in 5 older adults (22.5%; 95% CI: 20.0, 25.0) report an increase in loneliness during the pandemic. Those who were severely socially isolated had double the odds of feeling lonely. However, while unadjusted models suggest an increasingly positive correlation between social isolation and loneliness among older adults, associations in the fully adjusted model have weak and directionally variable correlations, suggesting that the relationship can be largely explained by other social and demographic characteristics. This may also explain why existing literature is divided on the relationship between social isolation and loneliness. During the pandemic, those who felt lonely were also more likely to strictly follow social isolation measures. The frequency of loneliness prior to the pandemic is a strong predictor of loneliness during COVID. Still, loneliness and isolation were only weakly correlated, even during this period of widespread increases in social isolation. The implications of social isolation and of loneliness for older adults’ wellbeing should be considered both jointly and independently, as they are not fully overlapping.

